# Metabolic reprogramming signature predicts prognosis and immune landscape in small cell lung cancer: MOCS2 validation and implications for personalized therapy

**DOI:** 10.3389/fmolb.2025.1592888

**Published:** 2025-05-16

**Authors:** Junyan Wang, Panpan Sun, Fan Zhang, Yu Xu, Shenghu Guo

**Affiliations:** ^1^ Medical Oncology Department, The Fourth Hospital of Hebei Medical University, Shijiazhuang, Hebei, China; ^2^ Department of Psychiatry, Hebei Province Rong-Jun Hospital, Baoding, Hebei, China; ^3^ Department of Immuno-Oncology, The Fourth Hospital of Hebei Medical University, Shijiazhuang, Hebei, China

**Keywords:** metabolic reprogramming, small cell lung cancer, prognosis, immune microenvironment, drug sensitivity

## Abstract

**Introduction:**

Small cell lung cancer (SCLC) remains a leading cause of cancer mortality worldwide, characterized by rapid progression and poor clinical outcomes, and the function of metabolic reprogramming remains unclear in SCLC.

**Methods:**

We performed multi-omics analysis using public SCLC datasets, analyzing single-cell RNA sequencing to identify metabolic reprogramming patterns between chemotherapy-resistant and sensitive samples. Bulk RNA sequencing from GSE60052 and cBioportal cohorts was used to identify metabolism-related gene modules through WGCNA and develop a Gradient Boosting Machine prognostic model. Functional validation of MOCS2, the top-ranked gene in our model, was conducted through siRNA knockdown experiments in SCLC cell lines.

**Results:**

Single-cell analysis revealed distinct metabolic reprogramming patterns between chemotherapy-resistant and sensitive samples. WGCNA identified a turquoise module strongly correlated with metabolic reprogramming (cor = 0.56, P < 0.005). The GBM-based prognostic model demonstrated excellent performance (C-index = 0.915) with MOCS2, USP39, SMYD2, GFPT1, and PRKRIR identified as the most important variables. Kaplan-Meier analysis confirmed significant survival differences between high-risk and low-risk groups in both validation cohorts (P < 0.001). *In vitro* experiments showed that MOCS2 knockdown significantly reduced SCLC cell proliferation, colony formation, and migration capabilities (all P < 0.01), confirming its crucial role in regulating SCLC cell biology. Immunological characterization revealed distinct immune landscapes between risk groups, and drug sensitivity analysis identified five compounds with significantly different response profiles between risk groups.

**Conclusion:**

Our study established a robust metabolism-based prognostic model for SCLC that effectively stratifies patients into risk groups with distinct survival outcomes, immune profiles, and drug sensitivity patterns. Functional validation experiments confirmed MOCS2 as an important regulator of SCLC cell proliferation and migration, providing valuable insights for treatment selection and prognosis prediction in SCLC.

## 1 Introduction

Lung cancer remains the leading cause of cancer-related mortality worldwide, with its histopathological classification primarily divided into small cell lung cancer (SCLC) and non-small cell lung cancer (NSCLC). SCLC constitutes approximately 14% of all cases with a male predominance (Wang et al., 2023; Rudin et al., 2021). This high-grade neuroendocrine carcinoma predominantly affects middle-aged and elderly populations with chronic tobacco exposure. Characterized by rapid proliferative capacity and poor clinical outcomes, untreated SCLC patients exhibit a median overall survival (OS) of merely 2–4 months from initial diagnosis (Petty and Paz-Ares, 2023).

Therapeutic resistance and complex drug tolerance in SCLC to insufficient understanding of its biological characteristics. Histogenetic origins of SCLC cells demonstrate remarkable diversity, including alveolar type 2 (AT2) cells, neuroendocrine (NE) cells, club cells, and basal cells. However, genomic analyses reveal that 75%–90% of SCLC cases exhibit inactivation of tumor suppressor genes TP53 and RB1 (Redin et al., 2024; Peifer et al., 2012; George et al., 2015). Molecularly targeted therapies based on transcriptional signatures (ASCL1/NEUROD1/YAP1 subtypes) show emerging clinical promise (Hu et al., 2024; Chen H. et al., 2023; Chen P. et al., 2023). Collectively, SCLC manifests profound intratumoral and intertumoral heterogeneity (Stewart et al., 2020; Rudin et al., 2019), a biological complexity that substantially complicates prognostic prediction and therapeutic response anticipation.

Tumor tissue needs to change its metabolic pathway to meet its material needs during rapid growth, a process called metabolic reprogramming. Metabolic alterations in tumors reshape their microenvironment, which in turn further drives metabolic changes, creating a reciprocal feedback loop that supports tumor progression. This aberrant metabolic pattern and the resulting tumor microenvironment contribute to immune cell dysfunction, further promoting tumor progression (Liu et al., 2024). Studies have confirmed that glucose, lipid, and amino acid metabolism are altered in SCLC to sustain rapid proliferation and survival (Huang et al., 2024). Targeting metabolism has been proposed as a promising approach in cancer research. Key metabolic enzymes such as LDH (Mezquita et al., 2018) and SLC7A11 (Iida et al., 2021) have prognostic value in cancer patients, and drugs targeting these enzymes have shown tumor-suppressive effects. However, research on metabolic reprogramming in SCLC remains limited.

Using public SCLC datasets, we constructed a prognostic model based on metabolism-related genes, analyzed the correlation between metabolic genes and tumor immunity, and assessed the potential drug resistance risks in patients with different metabolic risk profiles. This study provides new insights for treatment selection and prognosis prediction in SCLC.

## 2 Materials and methods

### 2.1 Data sources

Single-cell RNA sequencing (scRNA-seq) data were obtained from the Gene Expression Omnibus (GEO) database (https://www.ncbi.nlm.nih.gov/geo/) under accession number GSE138267 (Redin et al., 2024; Peifer et al., 2012; George et al., 2015). This dataset initially comprised 19 samples, including 8 chemotherapy-resistant and 11 chemotherapy-sensitive SCLC specimens. Following rigorous quality control assessment, we excluded three resistant samples and one sensitive sample due to insufficient quality metrics. The final analytical cohort consisted of 5 resistant and 10 sensitive samples, totaling 15 specimens for subsequent single-cell analyses. Bulk RNA sequencing data were derived from two independent sources: the GSE60052 dataset from GEO (Jiang et al., 2016), which contained 79 SCLC tumor samples and 7 adjacent non-tumor control samples, with 48 samples qualifying for inclusion in subsequent survival analyses after integrating clinical information; and the cBioportal database (https://www.cbioportal.org/) (Cerami et al., 2012), comprising 81 SCLC tumor samples, of which 77 were retained for prognostic analyses following survival data integration. To investigate metabolic reprogramming in SCLC, we curated a comprehensive gene set from multiple authoritative sources, including the GeneCards database (www.genecards.org) (selecting genes with relevance scores greater than 5 for metabolic processes) (Stelzer et al., 2016), the Kyoto Encyclopedia of Genes and Genomes (KEGG) metabolic pathways (https://www.genome.jp/kegg/) (Kanehisa et al., 2024), the REACTOME database (https://reactome.org/) metabolic pathway annotations (Milacic et al., 2023), and the Hallmark gene sets from the Molecular Signatures Database (MSigDB v5.2) (Liberzon et al., 2015), focusing on metabolism-related gene signatures.

### 2.2 Single-cell RNA-seq analysis

#### 2.2.1 Data preprocessing, quality control and integration

Single-cell RNA sequencing data were processed using the Seurat package (version 5.1.0) in R (Hao et al., 2024). Raw data from GSE138267 dataset were obtained as 10X Genomics format files and systematically processed to standardize file formats. Quality control metrics were calculated for each cell, including the number of detected genes, total read counts, percentage of mitochondrial, ribosomal, and hemoglobin genes. Cells were filtered using stringent thresholds (200 < nFeature_RNA <6,000, nCount_RNA <25,000, percent. mt < 10%, percent. HB < 1%) to retain high-quality single cells.

Data normalization was performed using Seurat’s NormalizeData function, followed by identification of the top 3,000 highly variable genes and data scaling. Principal component analysis was conducted with the optimal 30 principal components determined via elbow plot analysis. To mitigate batch effects, we employed the Harmony algorithm with 20 iterations, followed by dimensionality reduction using t-SNE and UMAP based on harmony-corrected principal components. Unsupervised clustering was performed using FindNeighbors and FindClusters functions with a resolution of 0.8.

#### 2.2.2 Cell type identification and characterization

Cell clusters were annotated based on the expression of canonical marker genes. We examined the expression patterns of key marker genes (Baine et al., 2020), including SCLC molecular subtype markers (ASCL1, NEUROD1), macrophage markers (VIM, HLA-DRB1, HLA-DQA1, BCL2A1), and NKT cell markers (ASCL1, CD1D, NCAM1). Four major cell populations were identified based on their gene expression profiles and visualized through uniform manifold approximation and projection (UMAP).

The cellular composition was compared between chemotherapy-resistant and chemotherapy-sensitive samples to identify potential differences in tumor cell distribution associated with treatment response. Differential gene expression analysis was performed between CTC type A and CTC type N cells to characterize their molecular distinction using the FindMarkers function in Seurat with parameters min. pct = 0.1 and logfc.threshold = 0.25.

#### 2.2.3 Pathway analysis and metabolic reprogramming assessment

Pathway enrichment analysis was conducted using the irGSEA package (Fan et al., 2024), which integrates multiple gene set enrichment methods (AUCell, UCell, singscore, ssgsea, JASMINE, and viper). We specifically examined hallmark gene sets from the MSigDB database to identify key pathways differentially activated between resistant and sensitive samples.

To evaluate metabolic reprogramming, we implemented a single-sample Gene Set Enrichment Analysis (ssGSEA) approach using a curated gene set derived from our integrated metabolic reprogramming gene collection. The ssGSEA scores were calculated for each cell, enabling us to quantify the degree of metabolic reprogramming at the single-cell level. The distribution of metabolic reprogramming scores was visualized across different cell types and compared between treatment response groups using Wilcoxon rank-sum tests. Cells were further stratified into “high” and “low” metabolic reprogramming groups based on the median score, and differential gene expression analysis was performed to identify genes associated with metabolic reprogramming phenotypes.

### 2.3 Weighted gene co-expression network analysis (WGCNA)

WGCNA was performed to identify gene modules associated with metabolic reprogramming in SCLC. The analysis was conducted using the WGCNA package in R on the GSE60052 dataset (Langfelder and Horvath, 2008), focusing on the metabolic reprogramming-related genes identified from our single-cell RNA-seq differential expression analysis.

Prior to network construction, data quality was assessed using the goodSamplesGenes function to remove genes with zero variance and detect outlier samples. Sample clustering was performed using hierarchical clustering with complete linkage method based on Euclidean distance to identify potential outliers. Following quality control, a signed co-expression network was constructed with an appropriate soft-thresholding power selected based on the scale-free topology fit index (R^2^) and mean connectivity criteria.

The network was constructed using the blockwiseModules function with the following parameters: TOMType = “unsigned” (for unsigned network topology), minModuleSize = 50 (minimum number of genes in a module), mergeCutHeight = 0.15 (threshold for merging similar modules), and maxBlockSize = 22,000 (for computational efficiency). This process enabled the identification of distinct gene modules, which were assigned different colors for visualization.

Module-trait relationships were evaluated by calculating Pearson correlations between module eigengenes (first principal component of each module) and the metabolic reprogramming scores derived from ssGSEA. The statistical significance of these correlations was determined using Student’s t-test with appropriate adjustment for multiple comparisons.

For key modules of interest, we calculated module membership (MM) values, which quantify the correlation between individual gene expression profiles and the module eigengene, as well as gene significance (GS) values, which represent the correlation between gene expression and metabolic reprogramming scores. The relationship between module membership and gene significance was analyzed to identify potential hub genes that might play essential roles in metabolic reprogramming processes in SCLC.

### 2.4 Machine learning model construction

To develop a robust predictive model for SCLC prognosis based on metabolic reprogramming gene signatures, we employed multiple machine learning approaches and ensemble strategies. The model development process involved data preparation, feature selection, model training, and validation using independent datasets.

Two independent SCLC cohorts were utilized: the GSE60052 dataset (n = 48) and the cBioportal dataset (n = 77). Common genes between datasets were identified, and batch effects were corrected using ComBat from the “limma” package. A combined training dataset was constructed by randomly sampling 60% of samples from each cohort, with the remaining samples reserved for validation.

We implemented and compared multiple survival prediction algorithms, including Random Survival Forest (RSF), Cox proportional hazards model with regularization (Lasso, Ridge, and Elastic Net), Gradient Boosting Machine (GBM), CoxBoost, Partial Least Squares Regression for Cox (plsRcox), Supervised Principal Components (SuperPC), and survival-Support Vector Machine (survival-SVM) (Sun et al., 2024). Additionally, we explored hybrid approaches by combining feature selection from one algorithm with model fitting from another.

For each algorithm, hyperparameters were optimized using cross-validation. For example, Random Survival Forest was configured with 1,000 trees and optimal node size determined through cross-validation. For Elastic Net, the alpha parameter was systematically varied from 0.1 to 0.9 to identify the optimal balance between ridge and lasso penalties.

Model performance was evaluated using Harrell’s concordance index (C-index) across both the training dataset and independent validation datasets. The C-index measures the model’s ability to correctly rank patient survival times, with values ranging from 0.5 (random prediction) to 1 (perfect prediction). Statistical comparisons between models were conducted to identify the most robust predictive approach for SCLC prognosis based on metabolic reprogramming signatures.

### 2.5 Risk stratification using optimal predictive model

To translate the metabolic reprogramming gene signature into a clinically applicable risk stratification system, we implemented the Gradient Boosting Machine (GBM) algorithm, which demonstrated superior performance among all tested models. Using this optimal model, patients were classified into high-risk and low-risk groups based on the median risk score threshold. The prognostic value of this risk stratification was evaluated using Kaplan-Meier survival analysis and log-rank tests. Model performance was assessed through Harrell’s C-index and time-dependent receiver operating characteristic (ROC) curves at 1-year, 3-year, and 5-year time points across all datasets. Additionally, variable importance analysis was conducted to identify the key molecular drivers within the metabolic reprogramming signature that most significantly contributed to outcome prediction.

### 2.6 Clinical analysis of metabolic risk model

To assess the clinical utility of our metabolic risk stratification model, we conducted comprehensive analyses of the relationship between risk groups and various clinical features. Clinical characteristics including gender, tumor stage (UICC stage, T stage, N stage, and M stage), and survival status were compared between high-risk and low-risk groups using chi-square tests. The distribution of these features was visualized through pie charts for each risk group, allowing for clear comparison of the proportional differences.

We further investigated the relationship between risk scores and tumor stage by examining the distribution of risk scores across different UICC stages. Violin plots combined with boxplots and jitter points were used to visualize these distributions, and Wilcoxon rank sum tests were performed to evaluate statistical significance between stage groups.

To evaluate the model’s ability to discriminate between early (stage I-II) and late (stage III-IV) disease, we constructed receiver operating characteristic (ROC) curves using logistic regression analysis. Finally, the prognostic value of our risk stratification system was assessed within specific clinical subgroups through Kaplan-Meier survival analysis, including separate analyses for early-stage (I-II) patients, late-stage (III-IV) patients, and different age groups (≤60 years and >60 years).

### 2.7 Prognostic value assessment through cox regression analysis

To determine the independent prognostic significance of our metabolic risk model, we performed comprehensive univariate and multivariate Cox regression analyses incorporating key clinical variables (age, gender, T stage, N stage, M stage, UICC stage) alongside the metabolic risk score. Forest plots were generated to visualize hazard ratios with 95% confidence intervals for each variable, enabling clear interpretation of their relative prognostic impact. The univariate analysis identified factors significantly associated with survival outcomes, while the multivariate analysis determined which variables maintained independent prognostic value when adjusted for other covariates.

Based on these findings, we developed an integrated nomogram incorporating the metabolic risk score with significant clinical parameters to provide individualized survival probability predictions at 1, 3, and 5 years. The nomogram’s performance was assessed through calibration curves comparing predicted versus observed survival probabilities, while decision curve analysis evaluated its clinical utility across various threshold probabilities.

### 2.8 Immunological characterization of metabolic risk groups

To explore the immunological differences between metabolic risk groups, we conducted comprehensive immune microenvironment analyses based on transcriptomic data. We applied ssGSEA to evaluate the enrichment of various immune-related pathways, with heatmap visualization highlighting significantly different immune pathways between high and low risk groups. The clear clustering pattern observed in the heatmap demonstrated distinct immune pathway activation states associated with our metabolic risk stratification.

For a more granular understanding of immune cell composition, we employed CIBERSORT to deconvolute the proportions of 22 immune cell types within each sample (Chen et al., 2018). Violin plots comparing immune cell fractions between risk groups revealed significant differences in multiple immune cell populations. Correlation analysis between risk scores and immune cell abundances identified key immune components associated with metabolic risk levels, providing insights into potential immune-metabolic interactions in SCLC. We also assessed 28 immune cell types using a published immune cell signature (Charoentong et al., 2017), further delineating the relationship between our metabolic risk model and tumor immune contexture. Finally, we examined correlations between key metabolic genes in our signature and specific immune cell populations, uncovering potential mechanistic links between metabolic reprogramming and immune regulation in SCLC.

### 2.9 Drug sensitivity analysis

To identify potential therapeutic strategies for SCLC patients with different metabolic risk profiles, we conducted a comprehensive drug sensitivity analysis. Using the pRRophetic package (Geeleher et al., 2014), we predicted the IC50 values of a wide range of anti-cancer compounds from the Cancer Genome Project (CGP) 2016 dataset (https://rdrr.io/github/xlucpu/MOVICS/man/cgp2016ExprRma.html) for each sample in our cohort. Drug response was compared between high-risk and low-risk metabolic groups using Wilcoxon rank-sum tests. Significant differences in predicted sensitivity were observed for several compounds, suggesting that our metabolic risk stratification could serve as a potential biomarker for drug response. For each drug showing statistically significant differences (p < 0.05), we generated detailed boxplots visualizing the distribution of predicted IC50 values across risk groups.

### 2.10 Experimental methods

#### 2.10.1 Knockdown model

Small cell lung cancer cell line H446 was obtained from the Cell Bank of the Chinese Academy of Sciences. Cells were cultured in RPMI-1640 medium supplemented with 10% fetal bovine serum and 1% penicillin-streptomycin, maintained at 37°C in a humidified incubator with 5% CO2. siRNA transfection was performed using Lipofectamine 3,000 reagent (Invitrogen, United States) according to the manufacturer’s instructions. MOCS2-targeted siRNAs and non-targeting control siRNA (si-NC) were synthesized by GenePharma (Shanghai, China). The sequences of MOCS2 siRNAs were as follows: siMOCS2-1: 5′-GGAUCAUACAGAUGAUAAAGU-3′; siMOCS2-2: 5′-GAUUUACUAUGUUGCAUAACU-3′. Cells were harvested 48 h post-transfection for subsequent experiments, and knockdown efficiency was verified by Western blot analysis.

#### 2.10.2 Western blot analysis

Total protein was extracted using RIPA lysis buffer containing protease and phosphatase inhibitors. Protein concentration was determined using the BCA Protein Assay Kit (Thermo Fisher Scientific, United States). Equal amounts of protein samples (30 μg) were separated on 12% SDS-PAGE gels and transferred to PVDF membranes (Millipore, United States). Membranes were blocked with 5% non-fat milk at room temperature for 1 h, then incubated overnight at 4°C with rabbit anti-human MOCS2 polyclonal antibody (1:1000 dilution, Abcam, United Kingdom) and mouse anti-human β-actin monoclonal antibody (1:5000 dilution, Proteintech, China). After washing three times with TBST, membranes were incubated with corresponding HRP-conjugated secondary antibodies (1:5000 dilution) at room temperature for 1 h. Protein bands were visualized using an enhanced chemiluminescence kit (ECL, Millipore, United States). Band intensities were analyzed using ImageJ software and normalized to β-actin as an internal reference.

#### 2.10.3 Colony formation assay

Transfected cells were seeded in 6-well plates at a density of 100 cells per well and cultured at 37°C with 5% CO2 for 14 days, with complete medium replacement every 3 days. After the culture period, medium was discarded, and cells were gently washed three times with PBS, followed by fixation with 4% paraformaldehyde for 15 min. After PBS washing, cells were stained with 0.1% crystal violet solution for 30 min. Following PBS washing and air-drying, images were captured using a stereomicroscope, and colonies containing more than 50 cells were counted. Three replicate wells were established for each group, and experiments were independently repeated three times.

#### 2.10.4 Cell migration assay (wound healing)

Transfected cells were seeded in 6-well plates at a density of 5 × 10^5^ cells per well and cultured until 80%–90% confluence. A sterile 200 μL pipette tip was used to create a straight scratch on the cell monolayer. After gentle washing with PBS to remove detached cells, serum-free medium was added to avoid the influence of cell proliferation on migration results. The scratch width at 0 h was photographed under an inverted microscope as a baseline, and cells were further cultured for 24 h before re-imaging the same position. Three replicate wells were established for each group, and experiments were independently repeated three times.

#### 2.10.5 Cell proliferation curve (CCK-8 method)

Transfected cells were seeded in 96-well plates at a density of 3 × 10^3^ cells per well, with five replicate wells per group. Measurements were taken at 0, 24, 48, 72, and 96 h. At each time point, 10 μL of CCK-8 reagent (Dojindo, Japan) was added to each well and incubated at 37°C for 2 h. Absorbance (OD value) was measured at 450 nm using a microplate reader. Cell proliferation curves were plotted with time as the abscissa and OD values as the ordinate. To eliminate the influence of cell density differences, OD values at each time point were normalized to growth rates relative to 0 h: relative proliferation rate = (OD value at each time point - OD value at 0 h)/OD value at 0 h. Experiments were independently repeated three times. Statistical analysis and graphing were performed using GraphPad Prism 8.0 software. Data are presented as mean ± standard deviation (Mean ± SD), and comparisons between groups were analyzed using one-way ANOVA. P < 0.05 was considered statistically significant.

## 3 Result

**FIGURE 1:**
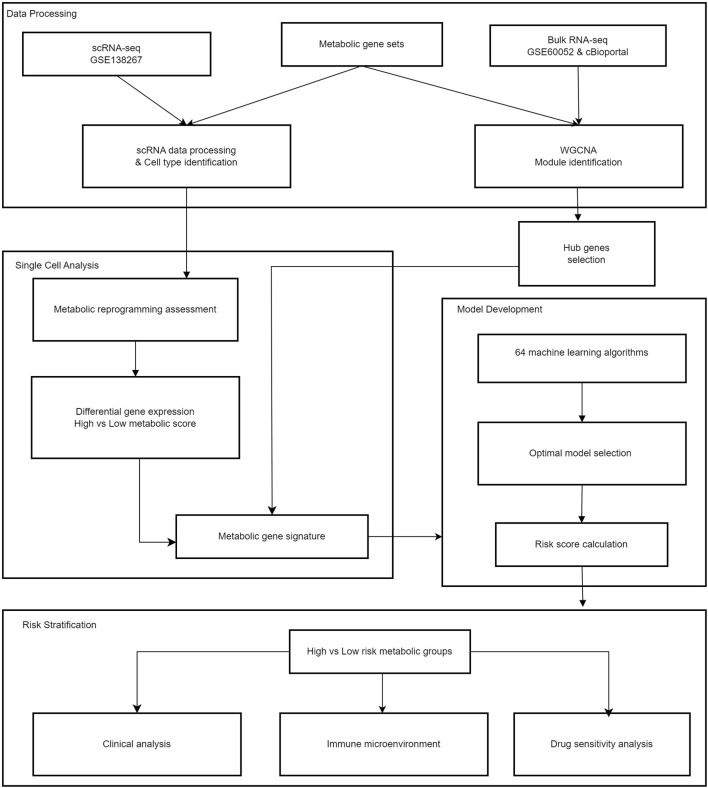
Study flowchart.

All analytical processes are illustrated in the flowchart (Figure 1).

### 3.1 Single-cell RNA sequencing analysis of circulating tumor cell subtypes and chemotherapy sensitivity

**FIGURE 2:**
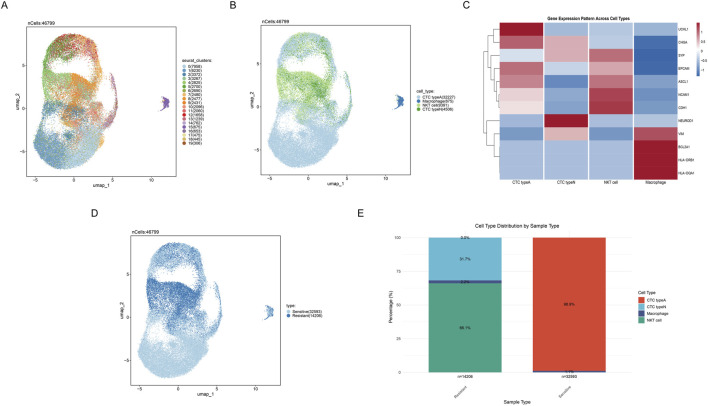
Cell clusters identifcation in scRNA-seq. **(A)** UMAP plot showing nineteen distinct cell clusters identified by unsupervised clustering analysis. **(B)** UMAP plot displaying four major cell populations identified based on gene expression profiles. Different colors represent different cell subgroups. **(C)** Heatmap depicting differential gene expression patterns across 4 cell types, where red and blue respectively indicate high and low gene expression levels. **(D)** UMAP plot distinguishing between sensitive and resistant cell populations. **(E)** Stacked bar plot showing the distribution proportions of cell subpopulations between sensitive and resistant samples.

Cells were divided into nineteen clusters and visualized through UMAP (Figure 2A). Based on canonical marker gene expression profiles, we successfully identified four major cell populations: CTC type A (UCHL1-high and ASCL1-high), CTC type N (NEUROD1-high), NKT cells, and macrophages (Figure 2B), thereby revealing the distribution patterns of various cell populations. The expression of cell cluster-specific marker genes was visualized in the heatmap, such as high expression of BCL2A1, HLA-DRB1, VIM and HLA-DQA1 in macrophages (Figure 2C). Additionally, we divided cells into two groups according to their sensitivity to chemotherapy response, where light blue indicates sensitive cells, and dark blue indicates resistant samples (Figure 2D). From the distribution, it can be seen that the sensitive and resistant samples show obvious distribution differences through UMAP (Figure 2D). Notably, comparative analysis of cellular composition between resistant and sensitive samples revealed differences in tumor cell distribution associated with chemotherapy response in SCLC microenvironment (Figure 2E). Results presented that sensitive samples exhibited a marked predominance of CTC type A cells (98.9%), whereas NKT cells (66.1%) predominated in resistant samples (Figure 2E).

### 3.2 Single-cell transcriptome reveals metabolic reprogramming patterns in SCLC

**FIGURE 3:**
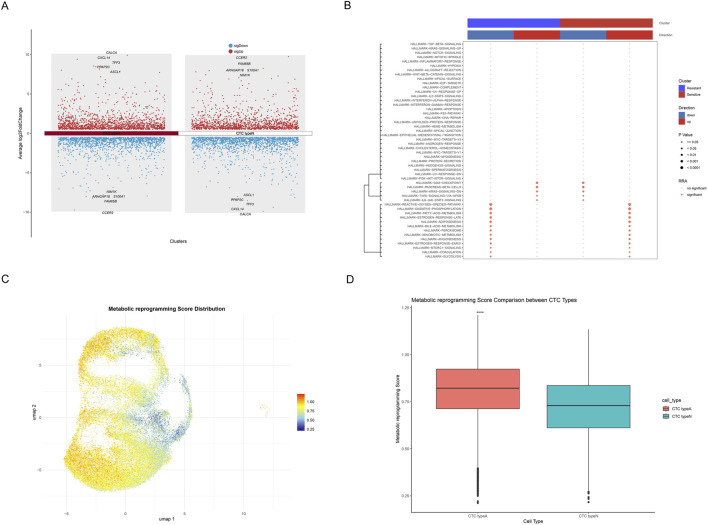
Metabolic Reprogramming Characterization of SCLC Single-cell Transcriptome. **(A)** Manhattan plot showing DEGs across CTC type A and CTC type N, with upregulated genes shown in red and downregulated genes in blue. Top 5 upregulated and top 5 downregulated genes are annotated. **(B)** Bubble plot of pathway analysis showing key pathways differentially activated between resistant and sensitive samples. The upper color bars indicate cluster identity (blue: resistant, red: sensitive) and regulatory direction (blue: downregulated, red: upregulated). Dot color indicates significance status (gray: no significant, red: significant), while dot size represents statistical significance (larger dots indicate smaller p-values). **(C)** UMAP plot showing the distribution of metabolic reprogramming scores, where a gradient from blue to red indicates increasing metabolic reprogramming scores. **(D)** Box plot indicating the comparison of metabolic reprogramming scores between CTC type A and CTC type N cells, with asterisks (****) indicating statistically significant differences (p < 0.0001).

The differentially expressed genes (DEGs) across CTC type A and CTC type N were systematically analyzed and visualized via manhattan plot (Figure 3A). Results showed that the top upregulated DEGs in CTC type A comprised CALCA, CXCL14, TFF3, PPAP2C, ASCL1 and the top downregulated DEGs including NIM1K, ARHGAP18, S100A1, FAM65B, and CCER2. While in CTC type N, CCER2, FAM65B, ARHGAP18, S100A1, NIM1K (upregulated) and ASCL1, PPAP2C, TFF3, CXCL14, CALCA (downregulated) were annotated. Furthermore, key pathways differentially activated between resistant and sensitive samples were identified by pathway analysis (Figure 3B). The bubble plot indicated that G2M-CHECKPOINT, PANCREAS-BETA-CELLS, KRAS-SIGNALING-DN, TNFA-SIGNALING-VIA-NFKB, and IL6-JAK-STAT3-SIGNALING were found to be upregulated in resistant samples and downregulated in sensitive samples (all *P* < 0.05). Moreover, results indicated that metabolism-related pathways including GLYCOLYSIS, FATTY-ACID-METABOLISM, OXIDATIVE-PHOSPHORYLATION, REACTIVE-OXYGEN-SPECIES-PATHWAY, BILE-ACID-METABOLISM, PEROXISOME, XENOBIOTIC-METABOLISM, MTORC1-SIGNALING, and ADIPOGENESIS were found to be upregulated in sensitive samples and downregulated in resistant samples (all *P* < 0.05). Similarly, signaling and growth-related pathways such as ANGIOGENESIS, ESTROGEN-RESPONSE-EARLY, ESTROGEN-RESPONSE-LATE, and COAGULATION showed the same patterns. These findings suggest that metabolic reprogramming, particularly involving energy production, lipid metabolism, and cellular detoxification processes, may contribute to chemotherapy resistance mechanisms. In addition, the metabolic reprogramming scores calculated through ssGSEA were visualized by UMAP (Figure 3C). Further comparative analysis demonstrated that CTC type A possesses significantly higher metabolic reprogramming scores than CTC type N (*P* < 0.0001, Wilcoxon rank-sum test, Figure 3D).

### 3.3 WGCNA analysis identifies key metabolic reprogramming modules in SCLC

**FIGURE 4:**
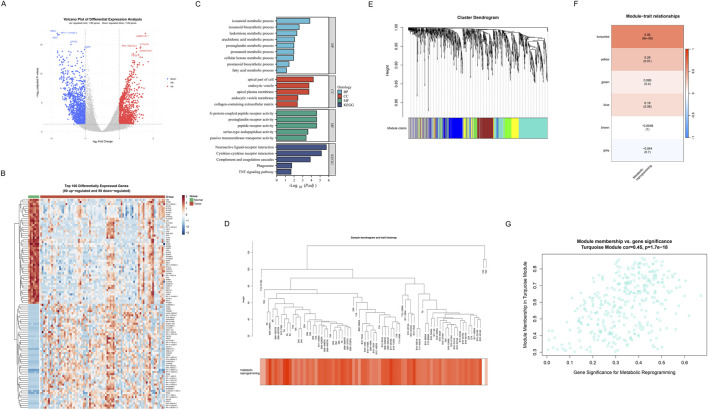
Identification of metabolic reprogramming-related genes in SCLC. **(A)** Volcano plot of differential expression analysis between SCLC samples and non-tumor control samples. Red represents upregulated genes (n = 1153), blue represents downregulated genes (n = 1100), and grey represents non-significant genes. The top 5 most significantly upregulated and downregulated genes are specially marked. **(B)** Hierarchical clustering heatmap of the top 100 DEGs (50 upregulated and 50 downregulated) across tumor and normal groups. The upper color bars indicate different groups (green: normal, red: tumor). Red indicates upregulated genes, and blue indicates downregulated genes. **(C)** GO and KEGG functional enrichment analysis results of DEGs. GO enrichment analyses include BP, CC, and MF. **(D)** Hierarchical clustering dendrogram of differential genes and heatmap of metabolic reprogramming score distribution. **(E)** Dendrogram revealing gene clustering relationships, with colored bands at the bottom representing 6 functional modules identified through the dynamic tree-cutting algorithm. **(F)** Module-trait correlation heatmap. Each row represents a co-expression module, and color intensity indicates Pearson correlation coefficients with metabolic reprogramming scores. **(G)** Scatter plot of gene significance for metabolic reprogramming versus module membership in the turquoise module (correlation = 0.45, p = 1.7e-18), identifying potential hub genes involved in metabolic reprogramming in SCLC.

Firstly, we displayed the DEGs between SCLC samples and non-tumor control samples through a volcano plot (Figure 4A). Compared with the non-tumor control samples, 1,153 genes were found upregulated, and 1,100 genes were downregulated in SCLC samples. Notably, the top five genes (HBA2, RP11-114H24.5, HBA1, SFTPC, AGER) were found to be significantly downregulated; whereas PABPC1P11, C17orf78, RP4-784A16.2, AC003102.3, and TIGD3 were observed to be significantly upregulated. To deeply analyze key regulatory genes, we then visualized the top 50 up- and downregulated genes with the most significant differences in a hierarchical clustering heatmap (Figure 3B). Results showed that the number of up-and downregulated gene expression was almost equal among these top 100 DEGs. Subsequently, GO and KEGG functional enrichment analyses were performed to investigate the distribution of these DEGs in biological processes (BP), cellular components (CC), and molecular functions (MF), as well as their potential roles in various biological pathways. Results indicated that in terms of BP, CC, and MF, the genes were primarily enriched in metabolic processes (icosanoid metabolic process), membrane-associated components (apical plasma membrane), and receptor-related molecular functions (G protein-coupled peptide receptor activity). Moreover, KEGG analysis revealed that the neuroactive ligand-receptor interaction pathway exhibited the most significant (Figure 3C). To systematically explore co-expression modules associated with metabolic reprogramming, WGCNA was performed on DEGs. The hierarchical clustering dendrogram demonstrated gene co-expression relationships and sample metabolic reprogramming score distribution (Figure 4D). Using the dynamic tree-cutting algorithm, six distinct functional modules were identified (Figure 4E). Notably, module-trait correlation analysis revealed that turquoise module showed strongest positive correlation (cor = 0.56, *P* < 0.005, Figure 4F). Further module membership analysis revealed that genes in the turquoise module showed significant positive correlation between GS and MM (cor = 0.45, *P* = 1.7e-18) (Figure 4G), strongly suggesting this module plays a core role in the metabolic reprogramming.

### 3.4 Development and validation of GBM-based prognostic prediction model

**FIGURE 5:**
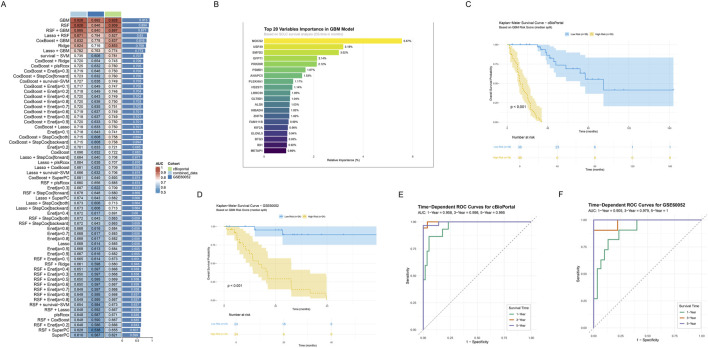
Construction and validation of Prognostic Signature Based on Integrated Machine Learning. **(A)** Performance evaluation heatmap of 64 machine learning algorithms. Rows represent different algorithms, and columns represent different datasets, including combined_data (light blue), cBioportal (green), and GSE60052 (dark blue). The color intensity indicates AUC values (red indicates higher AUC, blue indicates lower AUC). The extra last column represents the C-index scores across training and validation cohorts, and the top one represents the highest C-index. **(B)** Bar plot showing top 20 variables importance ranking in GBM model. **(C,D)** Kaplan-Meier survival curves in the two independent validation sets. Curves respectively showed survival differences between high (yellow) and low risk (blue) groups based on GBM risk scores for **(C)** cBioportal and **(D)** GSE60052. Shaded areas indicate 95% confidence intervals. Risk table below shows follow-up numbers at each time point. **(E,F)** Time-dependent ROC curves in the two independent validation sets. Green, red, and blue curves represent AUC values for 1-year, 3-year, and 5-year survival predictions. Curves respectively represent **(E)** cBioportal and **(F)** GSE60052.

Through systematic evaluation of eight machine learning algorithms and their various combinations, the GBM model demonstrated superior predictive performance (combined_data: AUC = 0.926, GSE60052: AUC = 0.892, cBioportal: AUC = 0.928, and C-index = 0.915, Figure 5A). Through variable importance analysis of the GBM model, the top 20 variables within the metabolic reprogramming signature were identified based on survival analysis in SCLC (Figure 5B). Among these, MOCS2, USP39, SMYD2, GFPT1 and PRKRIR showed the 5 highest relative importance.

To evaluate the prognostic value of risk stratification in GBM models, Kaplan-Meier survival analysis and time-dependent ROC curves analysis were conducted. Kaplan- Meier survival curves revealed significant prognostic differences between the groups stratified by risk scores calculated using GBM model both in the cBioportal and GSE60052 cohort (log-rank test, P < 0.001, Figures 5C,D). Notably, patients in the low-risk group demonstrated significantly prolonged OS compared to those in the high-risk group. Furthermore, time-dependent ROC curve analysis revealed consistent predictive performance across both cohorts. In the cBioPortal cohort (Figure 5E), AUC values were 0.958 (1-year), 0.998 (3-year), and 0.995 (5-year). Similarly, the GSE60052 cohort showed high AUC values of 0.905 (1-year), 0.979 (3-year), and 1 (5-year), indicating robust predictive efficiency (Figure 5F).

### 3.5 Clinicopathological characteristics of the low- and high-risk groups

**FIGURE 6:**
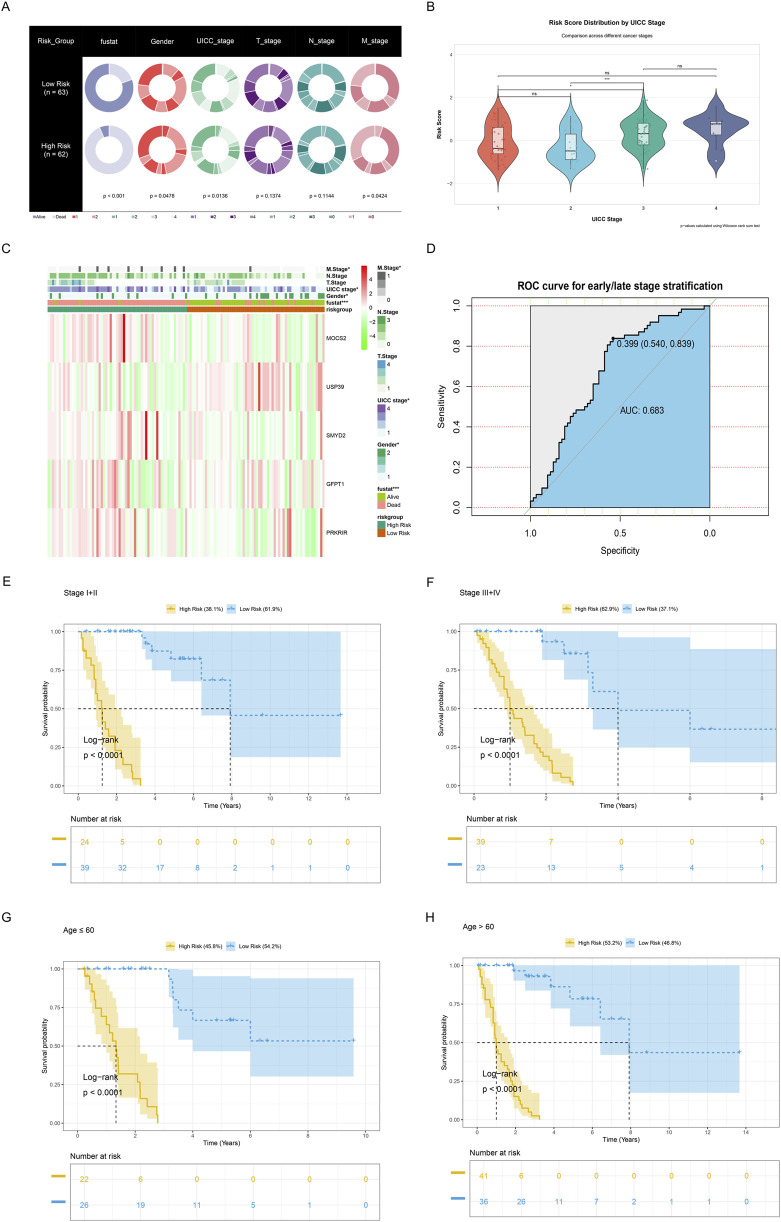
Clinicopathological characteristics of the low- and high-risk groups. **(A)** Pie charts demonstrating the distribution of clinical characteristics between low-risk (n = 63) and high-risk (n = 62) groups, including survival status (blue), gender (bright red), UICC stage (light green), T stage (purple), N stage (dark green), and M stage (dark red). **(B)** Violin plots combined with boxplots and jitter points illustrating risk score distribution by UICC stages. Color gradient from red (stage I) to dark blue (stage IV) reveals progressive risk score variations, with statistical significance levels denoted by * (*P* ≤ 0.05), ** (*P* ≤ 0.01), and *** (*P* ≤ 0.001). Non-significant differences are marked as “ns”, meaning *P* > 0.05. **(C)** Heatmap showing the expression levels of the five metabolic reprogramming-related genes and the distribution of clinicopathological variables between the low-risk and high-risk groups. *P < 0.05; **P < 0.01; ***P < 0.001. **(D)** ROC curve for early or late-stage stratification. **(E,F)** Kaplan-Meier survival analyses for **(E)** early- (I-II) or **(F)** late-stage (III-IV), revealing survival differences between high-risk and low-risk groups. Shaded areas indicate 95% confidence intervals. Risk table below shows follow-up numbers at each time point. **(G,H)** Kaplan-Meier survival analyses for patients aged ≤60 and ≥60 years, illustrating prognostic stratification across different age groups. Risk table below shows follow-up numbers at each time point.

A comprehensive evaluation of the metabolic risk stratification model’s clinical utility across diverse patient characteristics was conducted. Pie charts (Figure 6A) comparing clinical features, revealed the significant differences between high- (n = 62) and low-risk (n = 62) groups across clinical characteristics including survival status (*P* < 0.001), gender (*P* = 0.0478), UICC stage (*P* = 0.0136), and M stage (*P* = 0.0424). Furthermore, the distributions of risk scores across different UICC stages were analyzed. Results demonstrated statistically significant variations in risk score distribution across UICC stages (stages I vs. III, *P* < 0.05; stages II vs. III, *P* < 0.001), highlighting the model’s potential to capture disease progression (Figure 6B). Notably, patients at three to four stages had significantly higher risk scores than those at one to two stages. Additionally, five metabolic reprogramming-related genes expression and clinicopathological characteristics in both high-risk and low-risk groups were displayed (Figure 6C). Subsequently, the ROC curve demonstrated the model’s discriminative power between early and late-stage, with an AUC of 0.683. Furthermore, significantly higher survival probabilities were consistently observed in low-risk groups across various clinical subgroups via Kaplan-Meier survival curve, including early-stage (I-II), late-stage (III-IV), and age-stratified cohorts (≤60 and >60 years) (all *P* < 0.0001, Figures 6E–H).

### 3.6 Establishment and validation of a nomogram integrating clinical features

**FIGURE 7:**
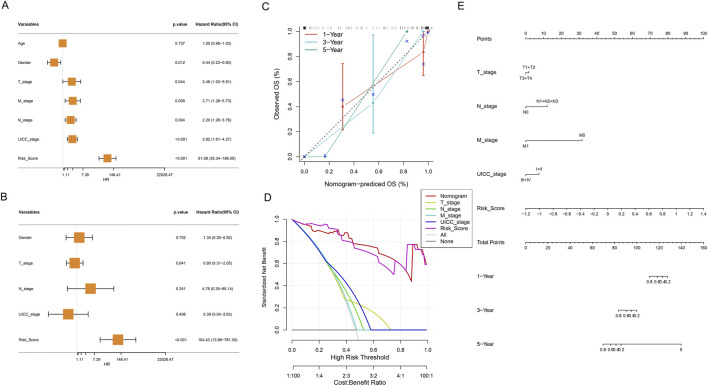
Comprehensive Cox Regression and Nomogram Analysis. **(A)** Forest plot of univariate cox regression analysis of clinical variables and metabolic risk score, presenting hazard ratios and statistical significance. **(B)** Forest plot of multivariate cox regression analysis revealing adjusted hazard ratios for clinical variables and metabolic risk score. **(C)** Calibration curve of the nomogram for 1, 3, and 5-year OS. Nomogram-predicted OS probabilities at different time points (red: 1-, blue: 3-, green: 5 - year), comparing predicted versus observed outcomes. **(D)** Decision curve analysis evaluating the clinical utility and net benefit of the prognostic model across different risk thresholds. **(E)** Nomogram integrating risk score and clinical characteristics.

To evaluate the independent prognostic value of our metabolic risk model, comprehensive cox regression analyses were performed incorporating clinical parameters with the metabolic risk score (Figures 7A,B). The forest plot of univariate cox regression analysis identified multiple significant prognostic factors including gender, T stage, N stage, M stage, UICC stage, and risk score as significant prognostic factors (all *P* < 0.05, Figure 7A). Further multivariate cox regression analysis confirmed the risk score as independent prognostic indicator through (*P* < 0.001, hazard ratio = 104.43, Figure 7B). Subsequently, we developed an integrated nomogram prediction model based on these prognostic factors including T stage, N stage, M stage, UICC stage, and risk score (Figure 7E). The nomogram calibration curves were then conducted to validate the predictive accuracy of the nomogram. Figure 7C showed alignment between predicted and observed survival probabilities across 1- and 3-year time points and highlighted the model’s robust predictive performance (Figure 7C). Furthermore, the nomogram was found to offer superior standardized net benefits within specific high-risk threshold ranges compared to individual clinical parameters alone through decision curve analysis, suggesting enhanced utility for clinical decision-making (Figure 7D).

### 3.7 Comprehensive immunological characterization revealing immune-metabolic interplay in SCLC through risk-group stratification

**FIGURE 8:**
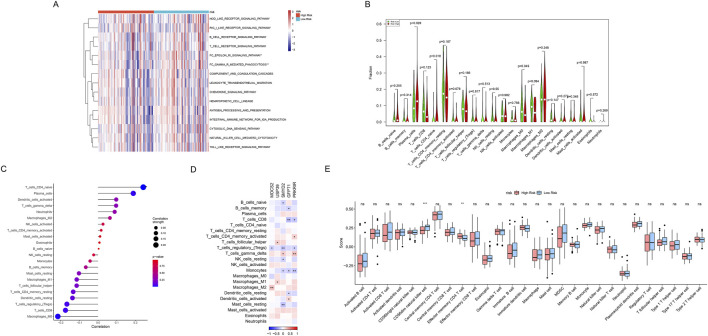
Comprehensive immunological characterization of metabolic risk groups. **(A)** Heatmap displaying the enrichment of immune-related pathways in high-risk (red) and low-risk (blue) metabolic groups. *P < 0.05, **P < 0.01. **(B)** Violin plot of infiltration proportion differences across 22 immune cells between (red) high- and (green) low-risk metabolic groups quantified by CIBERSORT algorithm. **(C)** Correlation scatter plot depicting relationship between risk scores and immune cell abundances, with point sizes representing correlation strength and color indicating statistical significance levels. **(D)** Correlation heatmap between metabolic risk score-related gene expression (columns) and immune cell populations (rows). Red indicates positive correlation, blue indicates negative correlation, and color intensity represents correlation strength. **(E)** Box plots comparing 28 gene-defined immune cell type abundances between high-risk (red) and low-risk (blue) metabolic groups. Box shows interquartile range, whiskers show 1.5 times interquartile range, outliers shown separately.

**FIGURE 9:**
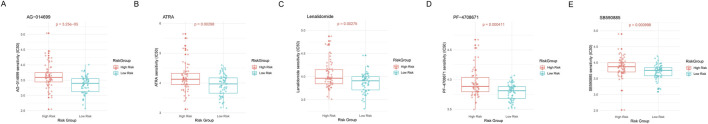
Drug sensitivity analysis. **(A–E)** Violin combined with the box plots showing IC50 value distribution of 5 key drugs between high- and low-risk metabolic groups. The Y-axis represents predicted IC50 values, with lower IC50 values indicating higher drug sensitivity. Red represents the high-risk metabolic group and blue represents the low-risk metabolic group.

The comprehensive immunological characterization reveals distinct immune landscapes between metabolic risk groups in SCLC. Differences in the activity of immune-related pathways were confirmed between the high-risk and low-risk metabolic groups, including FC_EPSILON_RI_SIGNALING_PATHWAY and FC_GAMMA_R_MEDIATED_PHAGOCYTOSIS (all *P* < 0.05, Figure 8A). We further used the CIBERSORT algorithm to calculate the abundance of 22 immune cells to further analyze the differences in specific immune cell infiltration between the high-risk and low-risk metabolic groups. It was found that high-risk metabolic groups had a significantly higher abundance of plasma cells (*P* = 0.029), T cells CD4 naïve (*P* = 0.018), while the low-risk group had a significantly higher abundance of macrophages M0 (*P* = 0.049, Figure 8B). Subsequently, correlation analysis between risk scores and immune cell infiltration (Figure 8C) indicated: (Wang et al., 2023): Significant positive correlations with T cells CD4 naive (*P* < 0.01), and plasma cells (*P* < 0.05); (Rudin et al., 2021); Significant negative correlations with macrophages M0 (*P* < 0.05). Moreover, potential mechanistic connections between metabolic reprogramming and immune regulation were revealed by correlation analysis between key gene expression and immune cell infiltration (Figure 8D). Finally, we quantified scores for 28 immune cell phenotypes (Figure 9D). Effector memory CD4 T cell (*P* < 0.01) showing a significant difference had higher expression levels in the high-risk metabolic group, with CD56dim natural killer cell (*P* < 0.001) showing higher expression in the low-risk metabolic group (Figure 8E).

### 3.8 Drug sensitivity analysis

We evaluated the practical utility of GBM risk classification for personalized treatment by analyzing drug sensitivity differences between metabolic risk groups. Five compounds demonstrated significant differential responses across risk strata (Figures 9A–E). The high-risk metabolic group showed distinct sensitivity patterns to several therapeutic agents: AG-014699 (PARP inhibitor, *P* = 3.25e-05, Figure 9A), ATRA (*P* = 0.00298, Figure 9B), Lenalidomide (immune modulator, *P* = 0.00275, Figure 9C), PF-4708671 (S6K1 inhibitor, *P* = 0.000411, Figure 9D), and SB590885 (BRAF inhibitor, *P* = 0.000998, Figure 9E). These findings establish a foundation for developing treatment strategies tailored to metabolic risk classification.

### 3.9 MOCS2 knockdown inhibits SCLC cell proliferation and migration

**FIGURE 10:**
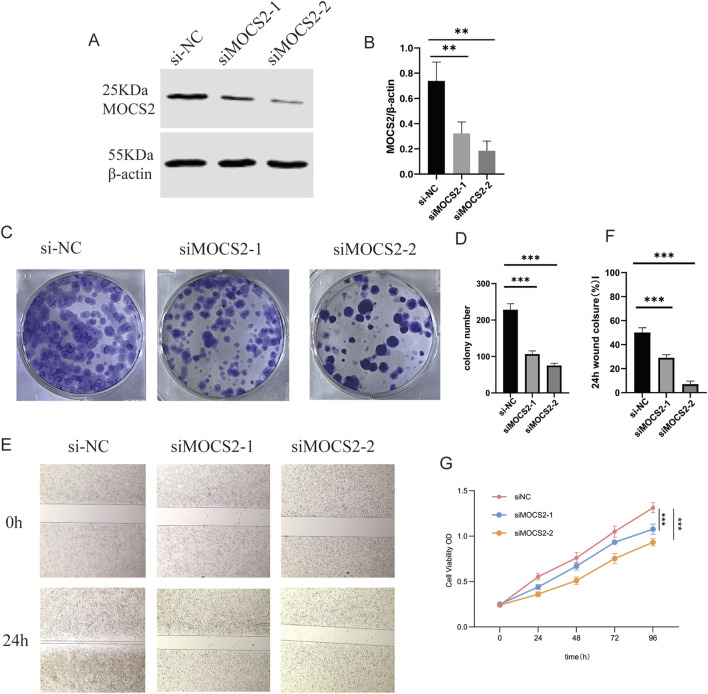
MOCS2 Knockdown Inhibits SCLC Cell Proliferation and Migration. **(A)** Western blot analysis showing changes in MOCS2 protein expression levels after siRNA transfection. **(B)** Quantitative analysis of MOCS2 protein expression levels, data presented as mean ± standard deviation (**P < 0.01). **(C)** Colony formation assay showing the effect of MOCS2 knockdown on cell colony formation ability. **(D)** Quantitative analysis of the colony formation assay, demonstrating significantly reduced colony numbers following MOCS2 knockdown (***P < 0.001). **(E)** Transwell migration assay showing the effect of MOCS2 knockdown on cell migration ability. **(F)** Quantitative analysis of the wound healing assay showing significantly reduced wound closure percentage at 24 h in MOCS2 knockdown cells (***P < 0.001). **(G)** CCK-8 proliferation assay showing the effect of MOCS2 knockdown on cell proliferation over 96 h (**P < 0.01, ***P < 0.001).

To validate the functional role of MOCS2, identified as one of the most important metabolism reprogramming-related genes in our GBM model, in SCLC, we conducted a series of *in vitro* functional experiments. First, we designed and synthesized MOCS2-specific siRNA and transfected it into SCLC cell lines. Western blot analysis showed that compared to the negative control siRNA (siRNA-NC) group, the MOCS2 protein expression level was significantly reduced in the si-MOCS2 treatment group, confirming the knockdown efficiency (Figures 10A,B).

Subsequently, we evaluated the impact of MOCS2 knockdown on the biological characteristics of SCLC cells through multiple methods. Colony formation assay results indicated that compared to the siRNA-NC group, the number of colonies formed in the si-MOCS2 group was significantly reduced (Figures 10C,D), suggesting that MOCS2 plays an important role in maintaining the survival and colony formation ability of SCLC cells.

In the Transwell migration assay, we found that the migration ability of cells in the MOCS2 knockdown group was significantly lower than that of the control group (Figures 10E,F), indicating that MOCS2 is involved in regulating the migratory properties of SCLC cells. Furthermore, CCK-8 proliferation assay showed that during the 96-h observation period, the proliferation rate of the si-MOCS2 transfection group remained consistently lower than that of the siRNA-NC control group (Figure 10G).

## 4 Discussion

### 4.1 Metabolic reprogramming as a key feature in SCLC progression and chemotherapy resistance

Our single-cell RNA sequencing analysis revealed distinctive metabolic reprogramming patterns between chemotherapy-resistant and chemotherapy-sensitive SCLC samples. The predominance of CTC type A cells in chemotherapy-sensitive samples and their significantly higher metabolic reprogramming scores compared to CTC type N cells suggests that specific metabolic configurations may influence therapeutic vulnerability in SCLC. Intriguingly, our pathway analysis demonstrated that several metabolism-related pathways—including glycolysis, fatty acid metabolism, oxidative phosphorylation, and xenobiotic metabolism—were significantly upregulated in chemotherapy-sensitive samples while being downregulated in resistant samples. This finding challenges the traditional Warburg effect paradigm (Warburg, 1956; Gatenby and Gillies, 2004), which suggests that increased glycolysis is predominantly associated with aggressive tumor behavior and therapeutic resistance. Instead, our results align with emerging evidence (Tavartkiladze et al., 2024) indicating that metabolic flexibility, rather than a specific metabolic program, may be crucial for therapy evasion in certain cancer contexts.

The identified metabolic differences between CTC type A (ASCL1-high) and CTC type N (NEUROD1-high) cells reflect the emerging recognition of molecular subtypes in SCLC. Our observation that CTC type A cells exhibit significantly higher metabolic reprogramming scores aligns with recent work by Zhang et al. (2022), who demonstrated distinct metabolic dependencies across SCLC subtypes. The predominance of ASCL1-high cells in chemotherapy-sensitive samples suggests that this subtype may possess metabolic vulnerabilities that could be therapeutically exploited, consistent with findings by Redin et al. (2024), who identified subtype-specific metabolic targets in SCLC. Furthermore, our results complement recent multi-omics analyses by Paunovic et al. (2024), who identified metabolic reprogramming as a key mechanism of acquired resistance in SCLC, though our single-cell approach reveals intrinsic metabolic differences that may predispose certain SCLC populations to chemotherapy sensitivity or resistance.

In summary, our findings establish metabolic reprogramming as a critical determinant of chemotherapy response in SCLC, with distinct metabolic signatures characterizing sensitive and resistant cell populations. These insights provide a foundation for developing metabolism-targeted therapeutic strategies that could potentially overcome or prevent chemotherapy resistance in this challenging malignancy.

### 4.2 Clinical relevance of the metabolic reprogramming signature

Our study identified a robust 5-gene metabolic signature (MOCS2, USP39, SMYD2, GFPT1, PRKRIR) that effectively stratifies SCLC patients into distinct prognostic groups. Unlike previous gene signatures in SCLC that focused primarily on cell cycle regulation or neuroendocrine differentiation (Wang et al., 2022), our signature specifically captures metabolic reprogramming processes, representing a novel perspective in SCLC risk stratification. Compared to the 10-gene signature developed by Xie et al. for SCLC (Xie et al., 2021), which showed moderate prognostic performance without validation dataset (C-index = 0.8), our metabolic model demonstrated superior predictive accuracy (C-index = 0.915), suggesting that metabolism-focused biomarkers may offer enhanced prognostic value in this disease context.

The clinical relevance of our metabolic risk classification is underscored by its significant correlation with key clinicopathological features and stage progression. This stage-dependent risk stratification aligns with recent findings by Shang et al. (2024), who observed increased metabolic pathway activation in advanced SCLC using metabolomic profiling. However, while their approach required specialized metabolomic platforms, our transcriptome-based signature provides a more clinically accessible tool for risk assessment. Furthermore, the independent prognostic significance of our metabolic risk score (HR = 104.43, P < 0.001) substantially exceeds that of previously reported gene signatures in SCLC, including the immune-related signature by Deng et al. (2022) (HR = 1.195).

Our nomogram integrating metabolic risk with clinical parameters demonstrated excellent predictive accuracy and enhanced clinical utility compared to conventional staging systems alone. This suggests that metabolic profiling addresses critical biological information not captured by traditional clinical assessments, consistent with observations by Wu et al. (2024) in their radiomics-based SCLC prognostic model. The consistent performance of our signature across diverse clinical subgroups further establishes its broad applicability as a prognostic tool, potentially enabling more personalized treatment approaches based on metabolic risk stratification in SCLC.

### 4.3 Immune-metabolic interplay in the SCLC microenvironment

Our comprehensive immunological characterization revealed distinct immune landscapes between high and low metabolic risk groups, suggesting a significant immune-metabolic interplay in SCLC. The high-risk metabolic group exhibited enrichment of specific immune pathways, including FC_EPSILON_RI_SIGNALING_PATHWAY and FC_GAMMA_R_MEDIATED_PHAGOCYTOSIS, indicating altered immune response patterns associated with metabolic reprogramming. This finding aligns with recent work by Xie et al. (2021), who identified immune-related pathways as prognostic determinants in SCLC, though our study uniquely links these immune signatures specifically to metabolic risk stratification.

The differential immune cell infiltration patterns observed between risk groups provide particularly compelling evidence for immune-metabolic interactions in SCLC. High-risk patients demonstrated significantly increased infiltration of plasma cells and naïve CD4 T cells, while low-risk patients showed enhanced macrophage M0 infiltration. This pattern differs from observations in NSCLC by Xiao et al. (2024), who found that increased macrophage infiltration correlated with worse prognosis, highlighting the unique immune microenvironment of SCLC. Our finding of increased effector memory CD4 T cells in high-risk patients, coupled with elevated CD56dim natural killer cells in low-risk patients, further suggests that metabolic reprogramming may influence both adaptive and innate immune responses in SCLC.

The significant correlations identified between our key metabolic genes and specific immune cell populations provide potential mechanistic insights into the immune-metabolic crosstalk in SCLC. Similar immune-metabolic interactions have been reported by Wang et al. in other cancers (Wang et al., 2024), where glycolytic activity influenced T cell function through lactate-mediated signaling. However, our study is the first to comprehensively map these correlations in SCLC, revealing potential therapeutic implications. For instance, the association between high-risk metabolic profiles and increased plasma cell infiltration suggests that targeting both metabolic pathways and B cell-mediated immunity might offer synergistic benefits in high-risk SCLC patients.

These findings extend beyond previous investigations by Jin et al. (2024), who examined PD-L1 expression in SCLC without considering the metabolic context. By integrating metabolic risk stratification with immune profiling, our study provides a more nuanced understanding of the immunosuppressive mechanisms in SCLC, potentially explaining the variable responses to immunotherapy observed in clinical practice (Wu et al., 2025). The distinct immune landscapes associated with different metabolic risk profiles suggest that metabolic reprogramming may serve as an upstream regulator of immune evasion in SCLC, offering novel perspectives for developing combination therapies that target both metabolic vulnerabilities and immune checkpoints to overcome treatment resistance in this challenging malignancy.

### 4.4 Limitations and future directions

While our study provides valuable insights into the role of metabolic reprogramming in SCLC progression and treatment response, several limitations warrant consideration. The retrospective nature of our analysis and the relatively modest sample size may limit the generalizability of our findings, necessitating prospective validation in larger, independent cohorts. Additionally, our reliance on transcriptomic data alone captures only one dimension of the complex metabolic landscape in SCLC. The computational inference of immune cell infiltration, while validated, cannot replace direct histological assessment or flow cytometry analysis of the tumor microenvironment. Our study also lacks functional validation of the identified metabolic genes, which is essential for establishing their causal roles in SCLC pathogenesis and therapy resistance. Future research directions should include *in vitro* and *in vivo* studies to verify the mechanistic contributions of MOCS2, USP39, SMYD2, GFPT1, and PRKRIR to metabolic reprogramming and immune modulation in SCLC. The potential therapeutic implications of our findings warrant exploration through preclinical studies of combinatorial approaches targeting both metabolic vulnerabilities and immune checkpoints, particularly in high metabolic risk SCLC models.

Development of metabolism-targeted therapies informed by metabolic risk stratification represents an exciting Frontier that may lead to more personalized treatment strategies. Integration of additional omics data, including proteomics and metabolomics, could further refine our current model by capturing post-transcriptional modifications and actual metabolite levels. Longitudinal studies examining changes in metabolic profiles during disease progression and in response to therapy would provide valuable insights into the dynamic nature of metabolic reprogramming in SCLC. Finally, investigating the potential interactions between metabolic reprogramming and other hallmarks of cancer, such as epigenetic alterations and DNA damage repair, may reveal more comprehensive therapeutic strategies for this challenging malignancy.

## 5 Conclusion

Our study reveals metabolic reprogramming as a critical determinant of SCLC progression and treatment response, identifying a 5-gene metabolic signature that effectively stratifies patients into distinct prognostic groups. This clinically robust risk model demonstrated superior predictive accuracy compared to conventional staging systems and correlated significantly with clinicopathological features and immune cell infiltration patterns. Our findings highlight the complex interplay between metabolic alterations and immune regulation in the SCLC microenvironment, offering potential explanations for variable therapeutic responses. The integration of metabolic risk assessment with drug sensitivity analysis provides a promising framework for personalized treatment selection, potentially guiding rational combination strategies targeting both metabolic vulnerabilities and immune checkpoints. By elucidating the central role of metabolic reprogramming in SCLC biology, our work contributes valuable insights that may ultimately improve patient stratification, treatment planning, and clinical outcomes in this challenging malignancy.

## Data Availability

The datasets presented in this study can be found in online repositories. The names of the repository/repositories and accession number(s) can be found in the article/supplementary material.
